# Multidrug-Resistant Bacteria on the Mobile Phones and Computer Keyboards of Healthcare University Students in Ghana

**DOI:** 10.1155/2021/6647959

**Published:** 2021-04-15

**Authors:** Michael Olu-Taiwo, Christian Afotey Laryea, David Kweku Mykels, Akua Obeng Forson

**Affiliations:** Department of Medical Laboratory Science, School of Biomedical and Allied Health Sciences, College of Health Sciences, University of Ghana, Legon, Ghana

## Abstract

Globally, mobile phones and computers (laptops and desktops) are indispensable part of human lives for communication, entertainment, and educational purposes. However, there are concerns about the increasing risk of bacterial contamination and antibiotic resistant trends from the surfaces of these devices. This study aims to assess bacterial contamination of mobile phones and computer keyboards and their resistant profile at the University of Ghana, Korle-Bu Campus, Accra. This was a cross-sectional study conducted from March to June 2017 with 240 swabs collected from the surfaces of mobile phones and computer keyboards used by healthcare students. Swabs were cultured on MacConkey, blood, and mannitol salt agar. Bacteria identification was performed with a standard bacteriological method. A total of 91 bacterial isolates were obtained from the devices, and they were tested against 9 commonly used antibiotics by the Kirby–Bauer disc method. The study revealed mobile phones and computer keyboards had contamination levels of 83.3% and 43.3%. Bacteria isolated included *Staphylococcus epidermidis* (25.4%), *Klebsiella* spp. (12.9%), *Staphylococcus aureus* (9.2%), *Escherichia coli* (6.7%), *Pseudomonas* spp. (5.4%), *Enterobacter* cloacae (2.1%), and *Enterobacter* spp. (1.7%). Overall, 91 bacterial isolates were highly resistant to ampicillin (96.7%) and tetracycline (75.8%) and moderately resistant to chloramphenicol (49.5%) with lower resistance to cefotaxime (18.7%), ceftadizime (14.2%), ciprofloxacin (25.3%), and gentamycin (24.7%). Additionally, 45.1% of isolates were multidrug resistant. Findings from this study revealed mobile phones and computer keyboards of healthcare students in the university were contaminated with pathogenic bacteria. Hence, frequent hand hygiene and disinfection of mobile phones and computer keyboard surfaces is encouraged to minimize the spread of resistant bacteria pathogens.

## 1. Introduction

Globally, mobile devices have emerged as a necessity for communication, entertainment, and educational purposes [1–3]. In 2018, the global digital agencies estimated worldwide mobile phone users to be over 5.11 billion [3]. Due to their relatively affordable prices and enhanced functions, mobile phone are found in most places in Ghana, and currently, there are over 19 million mobile-phone users and 9.28 million active mobile Internet users [3, 4]. Personal computers have likewise become a vital aspect of day-to-day activities in homes, offices, schools, hospitals, and laboratories [5, 6]. However, due to the immense benefits derived from usage of mobile phones and computers systems, their potential risk to human health may have been overlooked, since disinfection is virtually absent [4, 7]. The incessant handling of mobile phones, computer keyboards, and other electronic devices by various users exposes these devices to an array of pathogenic bacteria [7, 8]. Many of these pathogens have the capacity to survive on environmental surfaces; therefore, these could be potential sources of infection in humans [9, 10]. These devices have been found to act as formites for transmission of pathogenic agents such as *Staphylococcus aureus*, *Escherichia coli,* and *Pseudomonas* spp. [7, 11, 12]. Some of these pathogenic bacteria have been found to possess multidrug-resistance capacities [12, 13]. At present, antibiotic drug resistance has become a global issue that has led to an increase in morbidity and mortality, high treatment failures, and increased healthcare costs [13, 14].

Studies conducted in Ghana, Egypt, Ethiopia, and Pakistan on mobile phone and other devices have reported bacterial contamination prevalence of 100%, 100%, 100%, and 61.3%, respectively [1, 5, 7, 12]. Also, Tagoe et al.'s [12] study revealed 81.8% of bacterial isolates were found to be pathogenic and 100% of these bacterial isolates were resistant to ampicillin, cloxacillin, and penicillin. In another study in Nigeria [15], a bacterial contamination prevalence of 80% was reported and bacteria isolated included *Staphylococcus aureus* (53.6%), *Escherichia coli* (25.11%), and *Klebsiella* spp. (14.5%). However, in Ghana, there are limited data on bacterial contamination of mobile phones and computer keyboards with their associated antibiotic susceptibility patterns. Therefore, the present study was carried out to investigate the bacterial profile and their antibiogram from mobile phones and computer keyboards of healthcare students at the University of Ghana, Korle-Bu Campus.

## 2. Materials and Methods

### 2.1. Study Area and Design

This was a cross-sectional study conducted on the Korle-Bu Campus of the University of Ghana where students' mobile phones and computers (laptops and desktops) were randomly sampled from March to June 2017. The Korle-Bu Campus of the University of Ghana is located 3 kilometers from the Accra Central Business District and houses the Central Administration of the College of Health Sciences and the Schools of Medicine and Dentistry, as well as the School of Biomedical and Allied Health Sciences. Located within the same premises are the Korle-Bu teaching hospitals, which provide healthcare services to an estimated population of 3 million and serve as a referral hospital to a population of over 25 million [16].

### 2.2. Sample Collection

A total of 240 samples were randomly collected, and these comprised of 120 mobile phones and 120 computer keyboards. Of the 120 mobile phones sampled, 60 swabs were collected from students of the School of Biomedical and Allied Health Sciences, University of Ghana, and the other 60 swabs were from students of the School of Medicine and Dentistry of the University of Ghana. Also, of the 120 computer keyboards sampled, 10 swabs were from the IT Lounge, 20 swabs were from the SBAHS library, and the remaining 90 swabs were from students' personal laptops. Samples were collected using sterile cotton swabs moistened with 0.85% sterile saline solution and transported immediately to the SBAHS microbiology laboratory within 2 hr for bacteriological analysis.

### 2.3. Bacteriological Analysis

In the laboratory, swabs were inoculated onto blood agar plates (Oxoid, Cambridge, UK), MacConkey agar plates (Oxoid, Cambridge, UK), and Mannitol salt agar (Oxoid, Cambridge, UK) and incubated at 37^o^C for 18–24 hours for bacterial growth (34).

### 2.4. Identification of Bacterial Isolates

Identification of bacterial isolates was performed by first subculturing from primary culture plates to obtain pure culture colonies. Bacteria identification from pure culture plates was based on colonial morphology, and a representative colony on each plate was picked, Gram stained, and further tested using indole, methyl red, citrate, oxidase, and Voges–Proskauer test and urease, and coagulase tests [17]. An API 20 E identification system (bioMerieux SA, Marcy l‟Etoile, France) was also used to confirm the Gram-negative isolates.

### 2.5. Antimicrobial Susceptibility Test of Pathogenic Bacterial Isolates

Antibiotic susceptibility testing was performed for 91 pathogenic bacterial isolates excluding *Staphylococcus epidermidis* by the Kirby–Bauer disc diffusion method as recommended by the Clinical and laboratory and Standards Institute [18]. The procedure involved the preparation of an inoculum of 0.5 McFarland turbidity standards by transferring 2 to 3 colonies of an overnight culture of the test isolate on MacConkey agar (Oxoid, Cambridge, UK) into a sterile saline. A sterile cotton swab was then dipped into the 0.5 McFarland standard prepared inoculum and used to inoculate the entire surface of Mueller-Hinton agar plates (Oxoid, Cambridge, UK). Then, using a sterile forceps, the following commercially available antibiotics were placed on the streaked Mueller-Hinton agar plate: ampicillin (10 µg), ceftazidime (30 *µ*g), cefotaxime (30 *µ*g), ciprofloxacin (5 *µ*g), chloramphenicol (15 *µ*g), gentamicin (10 *µ*g), and tetracycline (30 *µ*g). Erythromycin (15 *µ*g) and rifampicin (5 ug) were included for *Staphylococcus aureus* isolates. The Mueller-Hinton agar plates were incubated aerobically at 37°C for 18–24 hr. The zone diameter of each of the antibiotics was measured with calipers and interpreted as per Clinical Laboratory and Standards Institute [18] recommendations. Control strains of *Pseudomonas aeruginosa* ATCC 27853 and *Escherichia coli* ATCC 25922 were used as controls to assist in the evaluation of the performance of the test.

### 2.6. Data Analysis

Data were entered into the Microsoft excel (2010) database and analyzed descriptively with SPSS version 20.0 (SPSS Inc., Chicago, IL). A frequency table was used to display numbers, percentages of isolates, antibiotic responses, and other variables. Chi square (*X*^2^) was used for comparison of any two categorical variables. Statistical significance was set at a *p* value of <0.05.

## 3. Results

### 3.1. Prevalence of Bacteria Isolated from Mobile Phones and Computer Keyboards

The overall prevalence of bacterial contamination on the mobile phones and computer keyboards was 83.3% (100/120) and 43.3% (52/120), respectively. There was a significant difference between the level of bacterial contamination on mobile phones and computer keyboards (*p* < 0.05) (Table 1). The most prevalent bacteria isolated from both the mobile phones and computer keyboards were *Staphylococcus epidermidis* (40.1% (61/150)), followed by *Klebsiella* spp. (20.4% (31/152)), *Staphylococcus aureus* (14.5% (22/152)), *Escherichia coli* (10.5% (16/152)), *Pseudomonas* spp. (8.6% (13/152)), and *Enterobacter cloacae* (3.3% (5/152)), and the least was *Enterobacter* spp. (2.6% (4/152)).

**Figure 1:**
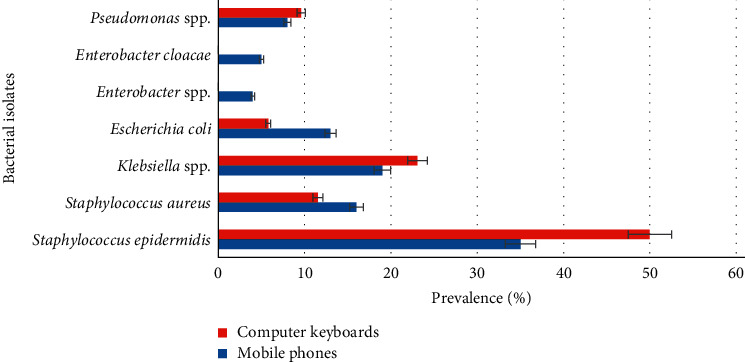
Prevalence of bacteria isolated from mobile phones and computer keyboards.

Both mobile phones and computer keyboards had high levels of *Staphylococcus epidermidis* (35% and 50%) and *Klebsiella* spp. (19% and 23%) (Figure 1). While low levels of *Staphylococcus aureus*, *Escherichia coli,* and *Pseudomonas* spp. was detected in both mobile phones and computer keyboards, *Enterobacter cloacae* and *Enterobacter* spp. were isolated from only mobile phones.

### 3.2. Distribution of Bacterial Isolates amongst Study Groups

Amongst allied health students and medical students, the most prevalent bacteria isolated were *Staphylococcus epidermidis* [16.7% (20/120) and 12.5% (15/120)], followed by *Klebsiella* spp. [10% (12/120)] from the allied students and *Staphylococcus aureus* [8.3% (10/120)] from medical students (Table 2). *Staphylococcus epidermidis* was also the prevalent bacteria on keyboards from students' laptops [10% (12/120)], library [7.5% (9/120)], and IT lounges [4.1% (5/120)], followed by *Klebsiella* spp. [6.7% (8/120) and 2.5% (3/120)] on keyboards of students' laptops and library (Table 2).

### 3.3. Antibiotic Susceptibility Patterns of Pathogenic Bacteria Isolated

**Figure 2:**
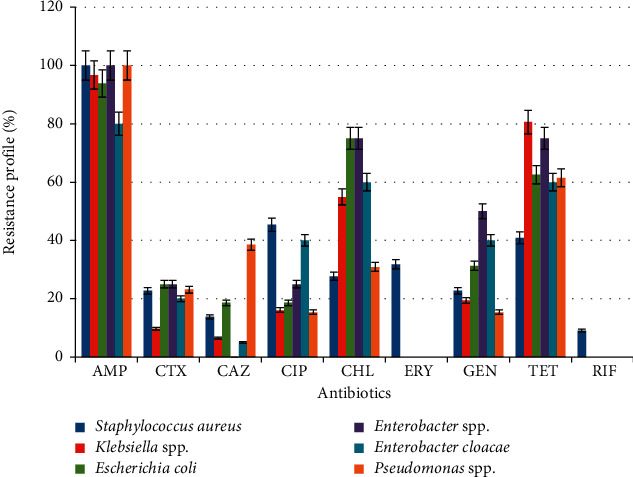
Resistance patterns of pathogenic bacteria isolated.

Ninety-one (91) pathogenic bacterial isolates were subjected to susceptibility testing. Overall, isolates showed high level of resistance to ampicillin (96.7%) and tetracycline (75.8%), a moderate resistance to chloramphenicol (49.5%), and a lower resistance to cefotaxime (18.7%), ceftazidime (14.2%), ciprofloxacin (25.3%), and gentamicin (24.7%) (Table 3). The resistant prevalence of the pathogenic bacteria isolated is shown in Figure 2.

Out of the 91 pathogenic bacterial isolates tested, 45.1% expressed multidrug resistance to the different tested antibiotics, with *Staphylococcus aureu*s (54.5%), *Klebsiella* spp. (29.3%), and *E*. *coli* (6.3%) showing varied levels of multidrug resistance (Table 4). In many cases, the resistance profile of the multidrug resistant isolates was highly varied within the same isolated species.

## 4. Discussion

Studies have shown that bacterial contamination of mobile phones, computer keyboards, and other hand-held devices may be involved in the spread of multidrug-resistant pathogenic bacteria [1, 2, 5]. In this study, there was an overall bacterial contamination level of 63.3% to mobile phones and computer keyboards, and bacterial contamination of 83.3% vs. 43.3% was observed for mobile phones vs. computer keyboards, respectively. Similar findings of 78.4%, 82.5%, 82.5%, 82.6%, 83.9%, and 86% have been reported for mobile phones sampled in Iran, Iraq, India, Ethiopia, Saudi Arabia, and Italy [5, 19–23]. In contrast to this study finding, a higher mobile phone bacterial contamination rate of 92%–100% has been documented in Slovakia, Turkey, Ethiopia, Saudi Arabia, and Ghana, respectively [12, 24–27]. However, lower mobile phone bacterial contamination rates of 29%, 33%, 34%, 40.6%, 58.3%, and 62% have been reported in Jordan, Brazil, Iran, India, Libya, and Nigeria [9, 28–32]. Contrary to this study finding that the computer keyboard bacterial contamination rate was 43%, higher contamination rates of 76% and 99% have been reported in Iran and India, respectively [33, 34]. However, lower bacterial contamination rates of 6.8% and 24% have been reported in the Netherlands and the United States of America, respectively [35, 36]. The varying bacterial contamination levels of mobile phones and computer keyboards in the various countries may be attributed to frequency of hand-washing practices and rates of cleaning mobile phones and computer keyboards [25]. In this study, *Staphylococcus epidermidis* (25.4%), *Klebsiella* spp. (12.9%), and *Staphylococcus aureus* (9.2%) were the predominant bacteria isolated. Similar findings of 28.4% for *Staphylococcus epidermidis* have been reported in Iraq [20]. Contrary to this study, a higher prevalence of 33.7% and 42.8% for *Staphylococcus epidermidis* have been documented in Jordan and Nigeria [28, 37]. However, a lower prevalence of 16% and 19% have been reported in studies in Saudi Arabia and Nigeria [9,38]. *Staphylococcus epidermidis* are normal flora of the skin and mostly associated with low virulence [25]. The high occurrence of *Staphylococcus epidermidis* on the devices may be due to the presence of the bacterium on the hands and skin. Comparably, findings of 12.7% and 14.4% for *Staphylococcus aureus* have been reported in Jordan and Ethiopia [25, 28]. In contrast to this study, higher prevalences of 16.2%, 20%, 30.6%, 35%, 39%, and 54.1% for *Staphylococcus aureus* have been reported in Saudi Arabia, Nigeria, Slovakia, Iraq, Libya, and India [9, 19, 24, 27, 32, 38]. *Staphylococcus aureus* is a common bacterium normally found on the skin and nasal region with an estimated 25% occurrence in healthy individuals [39]. *Staphylococcus aureus* have been associated with diseases that range from minor skin infections to more severe diseases, such as pneumonia, bacteremia, septicemia, and meningitis [39, 40]. Similar findings of 14.5% and 15.4% for *Klebsiella* spp. have been documented in Ethiopia and Nigeria [4–10]. In contrast to this study finding, lower prevalences of 4.8%, 6.9%, 6.9%, and 3.5% have been reported in studies in Nigeria, Iraq, Ethiopia, and Saudi Arabia [9, 12, 25, 27]. However, a higher prevalence of 33% has been reported in Slovakia [24]. *Klebsiella* spp. is one of the major causes of community and hospital-acquired infections, and it also has the propensity to disseminate mobile genetic elements [37, 41]. Similar findings of 6.5%, 5.5%, 6.8%, 8.57%, and 7.8% for *Escherichia coli* have been reported in Ethiopia, Saudi Arabia, Nigeria, Libya, and Iraq [9, 20, 25, 27, 32]. Contrary to this study, higher prevalences of 12.5% and 28.2% have been documented in studies in Egypt and Nigeria [1, 10]. However, a lower prevalence of 2.5% has been reported in a study in India [19]. The occurrence of *Escherichia coli* may be indicative of faecal contamination, probable due to minimal level of hand and mobile-phone hygienic practices. This bacterium is one of the most common etiological agents of diarrhoea, neonatal septicemia, urinary tract infections, bacteremia, and urosepsis [42]. It accounts for 80% of community-acquired urinary tract infections as well as 30% of nosocomial infections [43]. Presently, antibiotic-resistant bacteria have become a global public health issue to such an extent that it has led to high morbidity and mortality, long hospital stays, and higher treatment expenses [44, 45]. In this study, *Staphylococcus aureus* was 100% resistant to ampicillin. Comparable findings of 100% and 61.1% resistance to ampicillin have been reported in studies carried out in Nepal and Ethiopia [25, 46]. *Staphylococcus aureus* was 45.4% resistant to ciprofloxacin. A comparable resistant prevalence of 40.9% has been documented in a study in Bangladesh [47]. In contrast to this study's findings, lower resistance prevalences of 6.6%, 8.3%, 14.3%, and 19.3% have been documented in studies carried out in Nigeria, Rwanda, Iran, and Ethiopia [10, 25, 48, 49]. However, a higher resistant prevalence of 87.5% has been reported in a study carried out in Ethiopia [44]. *Staphylococcus aureus* was 22.7% resistant to gentamicin. Similar findings of 22.6% resistance to *Staphylococcus aureus* have been reported in a study performed in Ethiopia [25]. In contrast to this study, a higher resistant prevalence of 40.9% has been reported in a study conducted in Bangladesh [47]. However, a lower resistant prevalence of 15%, 12.1%, and 3.6% have been observed in studies conducted in Nepal, Nigeria, and Iran [10, 46, 49]. *Staphylococcus aureus* was 31.8% resistant to erythromycin. Comparable resistant prevalences of 23.2%, 36.4%, and 39.4% have been revealed in studies performed in Iran, Bangladesh, and Nigeria [9, 47, 49]. In contrast to this study finding, a higher resistant prevalence of 75% has been reported in a study carried out in Nepal [46]. In this study, *Klebsiella* spp. and *Escherichia coli* were 96.7% vs. 93.8% resistant to ampicillin. Comparable findings of 95% vs. 100% for *Klebsiella* spp. and *Escherichia coli* have been reported in Ethiopia [5]. However, in contrast to this study finding, lower resistance prevalences of 20% vs. 10.5% have been reported in Rwanda [48]. In this study, *Klebsiella* spp. and *Escherichia coli* were 19.4% vs. 31.3% resistant to gentamicin. Similar findings of 14% vs. 33% resistance prevalences have been documented in a study carried out in Ethiopia [5]. Contrary to these findings, higher resistant prevalences of 83.3% vs. 72.7% were reported in a study conducted in Nigeria [9]. In this study, *Klebsiella* spp. and *Escherichia* were 16.1% vs. 18.6% resistant to ciprofloxacin. Comparable findings of 13.3% vs. 31.6%, 8.1% vs. 19.1%, and 33% vs. 0% for *Klebsiella* spp. and *Escherichia coli* have been documented in studies from Rwanda, Nigeria, and Ethiopia [5, 10, 48]. The varying resistance prevalence may be due to different geographic locations or different policy on empirical treatment practices [25, 48]. In this study, the overall multidrug-resistance prevalence was 45.4%. A comparable multidrug-resistant prevalence of 37.9% has been reported in India [34]. In contrast, a higher multidrug-resistant prevalence of 69.9% has been documented in Ethiopia [25]. However, a lower multidrug-resistant prevalence of 9% is reported in Rwanda [48]. Multidrug-resistant bacterial strains may be attributed to indiscriminate and inappropriate use of antibiotics [25].

## 5. Conclusions

Mobile phones and computer keyboards of healthcare students in the university were found to be contaminated with pathogenic bacterial pathogens. Some of these bacterial pathogens were multidrug resistant. Mobile phones and computer keyboards could serve as a vehicle for transmission of antibiotic-resistant pathogens. Therefore, frequent hand hygiene and disinfection of mobile phones and computer keyboard surfaces is encouraged to minimize the spread of bacterial resistant strains.

### 5.1. Strength and Weaknesses

This study reports the bacterial contamination levels of mobile phones and computer keyboards in order to create awareness on the possible risk of these devises being vehicles for the transmission of antibiotic-resistant bacterial strains. To a greater extent, this will assist to disseminate information on resistant patterns of some multidrug-resistant bacteria pathogens. The study focused on bacterial contamination of mobile phones and computer keyboards with its associated resistant patterns. Decontamination of mobile phones and computer keyboards with 70% alcohol was not carried out to assess the contamination level after decontamination.

## Figures and Tables

**Table 1:** Prevalence of bacteria isolated from mobile phones and computer keyboards.

Bacterial isolates	Mobile phones	Computer keyboards	Total
	No. (%)	No. (%)	No. (%)
*Staph*. *epidermidis*	35 (29.2)^*∗*^	26 (21.7)	61 (25.4)
*Staph aureus*	16 (13.3)^*∗*^	6 (5.0)	22 (9.2)
*Klebsiella* spp.	19 (15.8)^*∗*^	12 (10.0)	31 (12.9)
*E*. *coli*	13 (10.8)^*∗*^	3(2.5)	16 (6.7)
*Enterobacter* spp.	4 (3.3)	—	4 (1.7)
*Enterobacter cloacae*	5(4.2)	—	5 (2.1)
*Pseudomonas* spp.	8 (6.7)	5 (4.2)	13 (5.4)
Total (%)	100 (83.3)^*∗*^	52 (43.3)	152(63.3)

^*∗*^ *p* value <0.05.

**Table 2:** Distribution of bacteria isolates amongst study areas.

	Mobile phones *n* = 120		Computer keyboards *n* = 120			Total (%)
Bacteria isolated	AHS (%)	MEDS (%)	STL (%)	L (%)	ITL (%)	
	(*n* = 60)	(*n* = 60)	(*n* = 90)	(*n* = 20)	(*n* = 10)	
*Staphylococcus epidermidis*	20(33.3)	15(25)	12 (13.3)	9(45)	5(50)	61 (25.4)
*Staphylococcus aureus*	6 (10)	10(16.7)	4(4.4)	1(5)	1(10)	22 (9.2)
*Klebsiella* spp.	12(20)	7(11.7)	8(8.9)	3(15)	1(10)	31 (12.9)
*Escherichia coli*	8(13.3)	5(8.3)	2 (2.2)	1 (5)	0	16 (6.7)
*Enterobacter* spp.	3(5)	1(1.7)	0	0	0	4 (1.7)
*Enterobacter cloacae*	2(3.3)	3 (5)	0	0	0	5 (2.1)
*Pseudomonas* spp.	5(8.3)	3 (5)	5 (5.6)	0	0	13 (5.4)

AHS: allied health students, MEDS: medical students, STL: student laptop, L: library, ITL: IT lounge.

**Table 3:** Antibiotic susceptibility patterns of pathogenic bacteria isolated.

Antibiotics	*Staphylococcus aureus*	*Klebsiella* spp.	*Escherichia coli*	*Enterobacter* spp.	*Enterobacter cloacae*	*Pseudomonas aeruginosa*	Total
	*n* = 22	*n* = 31	*n* = 16	*n* = 4	*n* = 5	*n* = 13	*n* = 91
	No. (%)	No. (%)	No. (%)	No. (%)	No. (%)	No. (%)	No. t(%)
AMP	22 (100)	30 (96.7)	15 (93.8)	4 (100)	4 (80.0)	13 (100)	88 (96.7)
CTX	5 (22.7)	3 (9.7)	4 (25)	1 (25.0)	1 (20.0)	3 (23.1)	17 (18.7)
CAZ	3 (13.8)	2 (6.5)	3 (18.6)	0 (0.0)	0 (0.0)	5 (38.5)	13 (14.2)
CIP	10 (45.4)	5 (16.1)	3 (18.6)	1 (25)	2 (40.0)	2 (15.4)	23 (25.3)
CHL	6 (27.7)	17 (54.9)	12 (75.0)	3 (75.0)	3 (60.0)	4 (30.9)	45 (49.5)
ERY	7 (31.8)	—	—	—	—	—	—
GEN	5 (22.7)	6 (19.4)	5 (31.3)	2 (50.0)	2 (40.0)	2 (15.4)	22 (24.7)
TET	9 (40.9)	25 (80.6)	10 (62.5)	3 (75.0)	3 (60.0)	8 (61.5)	69 (75.8)
RIF	2 (9.1)	—	—	—	—	—	—

AMP- ampicillin, CTX- cefotaxime, CAZ- ceftazidime, CIP- ciprofloxacin, CHL- chloramphenicol, ERY- erythromycin, GEN- gentamicin, TET- tetracycline, RIF- rifampicin.

**Table 4:** Prevalence of multidrug resistance among different bacterial isolates.

Bacteria	No. of isolates	Multidrug-resistance patterns
*Staphylococcus aureus*	3	AMP-CTX-CAZ-CIP-CHL-ERY-GEN-TET-RIF
	2	AMP-CTX-CIP-CHL-ERY-GEN-TET-RIF
	2	AMP-CTX-CIP-CHL-ERY-TET-RIF
	1	AMP-CIP-CHL-ERY-TET
	1	AMP-CIP-ERY-TET
	3	AMP-CIP-TET

*Klebsiella* spp.	3	AMP-CTX-CAZ-CIP-CHL-GEN-TET
	2	AMP-CTX-CIP-CHL-GEN-TET
	1	AMP-CIP-CHL-TET
	3	AMP-CIP-TET

*Escherichia coli*	3	AMP-CTX-CAZ-CIP-CHL-GEN-TET
	1	AMP-CTX-CHL-GEN-TET
	1	AMP-CHL-GEN-TET
	5	AMP-CHL-TET

*Enterobacter* spp.	1	AMP-CTX-CIP-CHL-GEN-TET
	1	AMP-CHL-GEN-TET
	1	AMP-CHL-TET

*Enterobacter cloacae*	1	AMP-CTX-CIP-CHL-GEN-TET
	1	AMP-CIP-CHL-GEN-TET
	1	AMP-CHL-TET

*Pseudomonas* spp.	2	AMP-CTX-CAZ-CIP-CHL-GEN-TET
	1	AMP-CTX-CAZ-CHL-GEN-TET
	1	AMP-CHL-TET

AMP- ampicillin, CTX- cefotaxime, CAZ- ceftazidime, CIP- ciprofloxacin, CHL- chloramphenicol, ERY- erythromycin, GEN- gentamicin, TET- tetracycline, RIF- rifampicin.

## Data Availability

The datasets used are available on reasonable demand.
